# *Insm1a* Is Required for Zebrafish Posterior Lateral Line Development

**DOI:** 10.3389/fnmol.2017.00241

**Published:** 2017-08-02

**Authors:** Yingzi He, Xiaoling Lu, Fuping Qian, Dong Liu, Renjie Chai, Huawei Li

**Affiliations:** ^1^ENT Institute and Otorhinolaryngology Department of Affiliated Eye and ENT Hospital, State Key Laboratory of Medical Neurobiology, Fudan University Shanghai, China; ^2^Key Laboratory of Hearing Medicine of NHFPC Shanghai, China; ^3^Key Laboratory for Developmental Genes and Human Disease, Ministry of Education, Institute of Life Sciences, Southeast University Nanjing, China; ^4^Jiangsu Key Laboratory of Neuroregeneration, Co-innovation Center of Neuroregeneration, Nantong University Nantong, China; ^5^Research Institute of Otolaryngology Nanjing, China; ^6^Institutes of Biomedical Sciences, Fudan University Shanghai, China; ^7^Shanghai Engineering Research Centre of Cochlear Implant Shanghai, China; ^8^The Institutes of Brain Science and the Collaborative Innovation Center for Brain Science, Fudan University Shanghai, China

**Keywords:** insulinoma-associated 1, zebrafish, posterior lateral line primordium, hair cell, Wnt/β-catenin signaling

## Abstract

Insulinoma-associated 1 (Insm1), a zinc-finger transcription factor, is widely expressed in the developing nervous system and plays important roles in cell cycle progression and cell fate specification. However, the functions of Insm1 in the embryonic development of the sensory system and its underlying molecular mechanisms remain largely unexplored. Here, through whole-mount *in situ* hybridization, we found that the zebrafish *insm1a* gene was expressed in the posterior lateral line (pLL) system, including both the migrating pLL primordium and the deposited neuromast cells. In order to decipher the specific roles of *insm1a* in zebrafish pLL development, we inhibited *insm1a* expression by using a morpholino knockdown strategy. The *insm1a* morphants exhibited primordium migration defects that resulted in reduced numbers of neuromasts. The inactivation of *insm1a* reduced the numbers of hair cells in neuromasts, and this defect could be a secondary consequence of disrupting rosette formation in the pLL primordium. Additionally, we showed that *insm1a* knockdown decreased the proliferation of pLL primordium cells, which likely contributed to these pLL defects. Furthermore, we showed that loss of *insm1a* resulted in elevated Wnt/β-catenin signaling and downregulation of Fgf target genes in the primordium. *Insm1a* knockdown also perturbed the expression patterns of chemokine signaling genes. Taken together, this study reveals a pivotal role for *Insm1a* in regulating pLL development during zebrafish embryogenesis.

## Introduction

The lateral line of zebrafish is a mechanosensory system that is implicated in several behaviors, including water movement detection and localization, schooling, predator avoidance, prey detection and capture, and so on. This system is composed of the anterior lateral line, located on the head, jaw, and opercle, and the posterior lateral line (pLL) that is distributed in a stereotypical pattern over the surface of the trunk and tail. The pLL develops from the pLL primordium (pLLp), which consists of ~100 cells and arises at the cranial placode just posterior to the otic vesicle. During early embryonic stages, the pLLp migrates caudally along the horizontal myoseptum of the embryo between 22 and 48 h post-fertilization (hpf), depositing the sensory organs called neuromasts at regular intervals and ultimately generating five or six neuromasts on the trunk and two or three terminal neuromasts at the tail (Ghysen and Dambly-Chaudiere, 2004, 2007). Each neuromast is composed of mechanosensory hair cells in the center and supporting cells at the periphery (Dambly-Chaudière et al., 2003; Ghysen and Dambly-Chaudiere, 2004). The neuromast hair cells are functionally and morphologically related to the hair cells in the vertebrate inner ear (Whitfield, 2002; Nicolson, 2005).

Morphogenesis of the pLL is a complicated and highly regulated set of processes, including pLLp directional migration, proliferation, neuromast deposition, and hair cell differentiation. All of these processes are tightly controlled by a network of signaling pathways, including chemokine, canonical Wnt/β-catenin, and fibroblast growth factor (Fgf) signaling (Aman and Piotrowski, 2008, 2009, 2011; Ma and Raible, 2009). Canonical Wnt/β-catenin and Fgf signaling are essential for pLL morphogenesis during embryonic development, and misregulation of these signaling pathways through genetic or pharmacological modulation results in multiple pLL malformations due to defects in the processes of cell proliferation, cell fate determination, and cell differentiation (Aman and Piotrowski, 2008; Lecaudey et al., 2008; Aman et al., 2011; McGraw et al., 2011). Additionally, knockdown of *cxcl12a* or either of its receptors *cxcr4b* or *cxcr7b*, which are expressed in the leading or trailing part of the pLLp, respectively, leads to strong migration defects (Li et al., 2004; Haas and Gilmour, 2006; Dambly-Chaudiere et al., 2007). Accumulating evidence has shown that much of the signaling regulation is accomplished by the sequential expression of cascades of transcription factors in which the activities of one set of transcription factors control some signaling pathways as well as the expression of other sets of transcription factors that in turn regulate signaling pathways. However, the specific transcription factors involved in pLL development are still largely uncharacterized.

Insulinoma-associated protein 1 (Insm1 or IA-1) is a conserved zinc-finger transcription factor that was originally cloned from a human insulinoma subtraction library (Goto et al., 1992). *Insm1* mRNA is abundantly expressed in the developing central and peripheral nervous systems, in the olfactory epithelium, and in the endocrine cells (Gierl et al., 2006; Farkas et al., 2008; Wildner et al., 2008; Rosenbaum et al., 2011), and several lines of evidence indicate that *insm1* expression can be controlled by the basic helix-loop-helix transcription factors (Breslin et al., 2003; Mellitzer et al., 2006). Insm1 is an important transcriptional repressor that performs multiple functions in early embryonic neurogenesis (Breslin et al., 2002). Deletion of the *insm1* gene in the cortex and olfactory epithelium leads to fewer basal progenitors and consequently to fewer neurons (Farkas et al., 2008; Rosenbaum et al., 2011), and global *insm1* deletion in mice results in early embryonic lethality (Gierl et al., 2006). In the zebrafish sensory system, *insm1a* is necessary for photoreceptor differentiation, and it regulates cell cycle progression in retinoblasts and functions upstream of the bHLH transcription factors during retinal development (Forbes-Osborne et al., 2013). In the embryonic mouse ear, *insm1* is expressed in delaminating progenitor cells and in nascent spiral and vestibular ganglia (SVG) neurons (Lorenzen et al., 2015). *Insm1* is also expressed in nascent outer hair cells of the embryonic mouse cochlea, but not in inner hair cells, and *insm1* ablation in mice results in fewer SVG neurons and reduced proliferation of delaminated progenitors (Lorenzen et al., 2015). Despite knowledge of its specific roles in some contexts, the role of insm1 in zebrafish pLL development has not yet been studied.

Here, we show that the zebrafish *insm1a* gene is expressed in the developing pLL system. By knocking down *insm1a* with its specific morpholino, we demonstrate that disrupting the expression of *insm1a* results in a disorganized pLL pattern with fewer numbers of neuromasts along the body and a lack of hair cells within the neuromasts. Loss of *insm1a* decreased the normal proliferation of pLLp cells, and the transcription of *lef1* and *axin2*, which are known Wnt-activated transcription factors that are important for pLL morphogenesis, were upregulated in *insm1a*-deficient primordia. Importantly, our results also suggest that chemokine signaling in the pLL might be regulated by *Insm1a*. Taken together, this work represents the first report on the function of *Insm1a* in regulating some signaling pathways that control pLL formation during zebrafish embryogenesis.

## Materials and methods

### Zebrafish lines and maintenance

All zebrafish animal procedures were carried out following the institutional guidelines approved by the Institutional Animal Care and Use Committee of Fudan University, Shanghai. The pLLp and hair cells were visualized using the *tg*(*cldnb*:lynGFP) line and the *tg*(*Brn3c*:GFP) line, respectively. Embryos were obtained by natural spawning and developed at 28.5°C on a 14 h light:10 h dark cycle. Embryos were staged as previously described (Kimmel et al., 1995).

### Micro injections of morpholinos and mRNA

For morpholino oligonucleotide (MO) knockdowns, embryos were injected with MOs at the one-cell to two-cell stage. All MOs were synthesized by GeneTools, LLC (Philomath, OR, USA). The MO injections were 6 ng of *insm1a*-MO (5′-AAATCCTCTGGGCATCTTCGCCAGC-3′) or 6 ng of the standard control-MO, (5′-CCTCTTACCTCAGTTACAATTTATA-3′). To avoid off-target effects, 9 ng of antisense *p53*-MO (5′-GCGCCATTGCTTTGCAAGAATTG-3′) was co-injected with *insm1a*-MO. *Insm1a* mRNA was transcribed with the mMESSAGE Machine Sp6 Kit (Ambion, Austin, TX, US) according to the manufacturer's instructions. For mRNA injections, 200 pg of *insm1a* mRNA was injected into one-cell-stage embryos and incubated at 28°C until the desired stages.

### Immunohistochemistry

Embryos were fixed overnight at 4°C in 4% paraformaldehyde (PFA) and were washed with PBT-2 (PBS containing 0.5% Triton X-100) three times followed by incubation in blocking solution for 1 h at room temperature. Primary antibodies were then added to this blocking solution and incubated overnight at 4°C with rocking. The following antibodies were used as primary antibodies: anti-GFP (1:1000 dilution; Abcam, Cambridge, UK), anti-cleaved caspase-3 (1:500 dilution; Cell Signaling Technology Inc., Danvers, MA, USA), and anti-E-cadherin (1:200 dilution; BD Biosciences,USA). After three washes with PBT-2, Alexa Fluor 488– and 594–conjugated secondary antibodies (Jackson Immuno Research, West Grove, PA, USA) were all used at 1:500 dilution and incubated overnight at 4°C with rocking. Nuclei were labeled with 4,6-diamidino-2-phenylindole (DAPI; 1:1000 dilution; Invitrogen, Carlsbad, CA, USA) for 20 min at room temperature. For image collection, Z-sections were taken at 1 μm intervals through the depth of the primordium/neuromast. Maximum-intensity projections were generated for analysis, and images were processed using Photoshop software (Adobe). Cell counts were performed at the time of imaging by viewing the images under a fluorescence microscope (Eclipse; Nikon Instruments) using a 40× objective.

### BrdU injection and immunohistochemistry

For proliferation analysis, control and *insm1a* morphant embryos were dechorionated and incubated with a 15 mM solution of BrdU (Sigma-Aldrich, St. Louis, MO, USA). The embryos were returned to 28°C and collected at 1 h post treatment. The embryos were anesthetized with ethyl 3-aminobenzoate methanesulfonate salt (MS-222, Tricaine, Sigma-Aldrich). The embryos were immunostained as described above, with the addition of 2N HCl for 0.5 h at 37°C prior to blocking in 10% normal goat serum. Following incubation with the monoclonal anti-BrdU primary antibody, embryos were washed three times with PBT-2 and then incubated with the secondary antibody for 1 h at 37°C. Fluorescently labeled embryos were imaged with a Leica confocal fluorescence microscope (TCS SP8; Leica, Wetzlar, Germany). Maximum-intensity projections were generated for analyses, and images were processed using Photoshop software (Adobe).

### Whole-mount *in situ* hybridization

Regular whole-mount *in situ* hybridization (WISH) of zebrafish embryos was performed as previously described (Thisse and Thisse, 2008). Briefly, the embryos were depigmented with 1-phenyl-2-thiourea (Sigma-Aldrich), euthanized in MS-222, and fixed in 4% PFA at 4°C overnight. Fixed embryos were then washed in PBST (PBS with 0.1% Tween-20) and stored in 100% methanol at −20°C for dehydration. For *in situ* hybridization, embryos were rehydrated in a graded methanol series and washed three times with PBST. To permeabilize the embryos, proteinase K (20 μg/ml in PBST) was added, and the embryos were re-fixed in 4% PFA for 20 min. After washing in PBST, the embryos were prehybridized at 65°C for ≥2 h in hybridization buffer. The labeled probes were added to the hybridization buffer at 65°C overnight. Embryos were washed through a graded SSC series at 65°C before blocking for a minimum of 1 h in blocking buffer (Roche). Embryos were incubated overnight at 4°C with an anti-digoxigenin-AP Fab fragment (Roche) diluted 1:4000 in blocking buffer. The following day, the embryos were washed 4 × 30 min with 2 mg/mL BSA in PBST and equilibrated in NTMT buffer (0.1M Tris pH 9.5, 0.05M MgCl_2_, 0.1M NaCl, and 0.1% Tween-20). The embryos were then stained with BM purple AP substrate (Roche) in the dark. The color reaction was stopped by washing with PBST, and the embryos were then re-fixed in PFA, cleared in 75% glycerol/PBS, and imaged on a bright-field microscope (Eclipse Ti-U; Nikon Instruments, Melville, NY, USA). Sites of binding were identified as blue-black dots.

### TUNEL staining

The TUNEL (Terminal deoxynucleotidyl transferase-mediated dUTP nick end labeling) cell death assay was performed as described previously (Cai et al., 2016) using the *In Situ* Cell Death Detection Kit, Fluorescein (Roche, Nutlet, NJ, USA; cat. no. 11684795910).

### Western blot analysis

Embryos collected at the indicated times were lysed in RIPA buffer. The proteins were separated by SDS-PAGE and then transferred onto PVDF membranes (Immobilon-P; Millipore, Bedford, MA, USA). The membranes were then blocked with 5% nonfat dried milk in TBST (20 mM Tris-HCl (pH 7.5), 500 mM NaCl, and 0.1% Tween-20) for 1 h at room temperature and subsequently incubated with the corresponding primary antibody overnight at 4°C. The next day, the blots were washed three times with TBST (5 min/wash) and subsequently blotted with horseradish peroxidase-conjugated secondary antibody for 1 h at room temperature. The reactions were detected using ECL Prime Western Blotting Detection Reagent (GE Healthcare, Wauwatosa, WI, USA).

### Statistical analysis

All data were analyzed using GraphPad Prism (version 6). Statistical analyses were performed by two-tailed Student's *t*-test (see figure legends for details), and a *p*-value < 0.05 was considered statistically significant. All data are presented as the mean ± s.e.m.

## Results

### *Insm1a* is expressed in the zebrafish lateral line system

**Figure 1:**
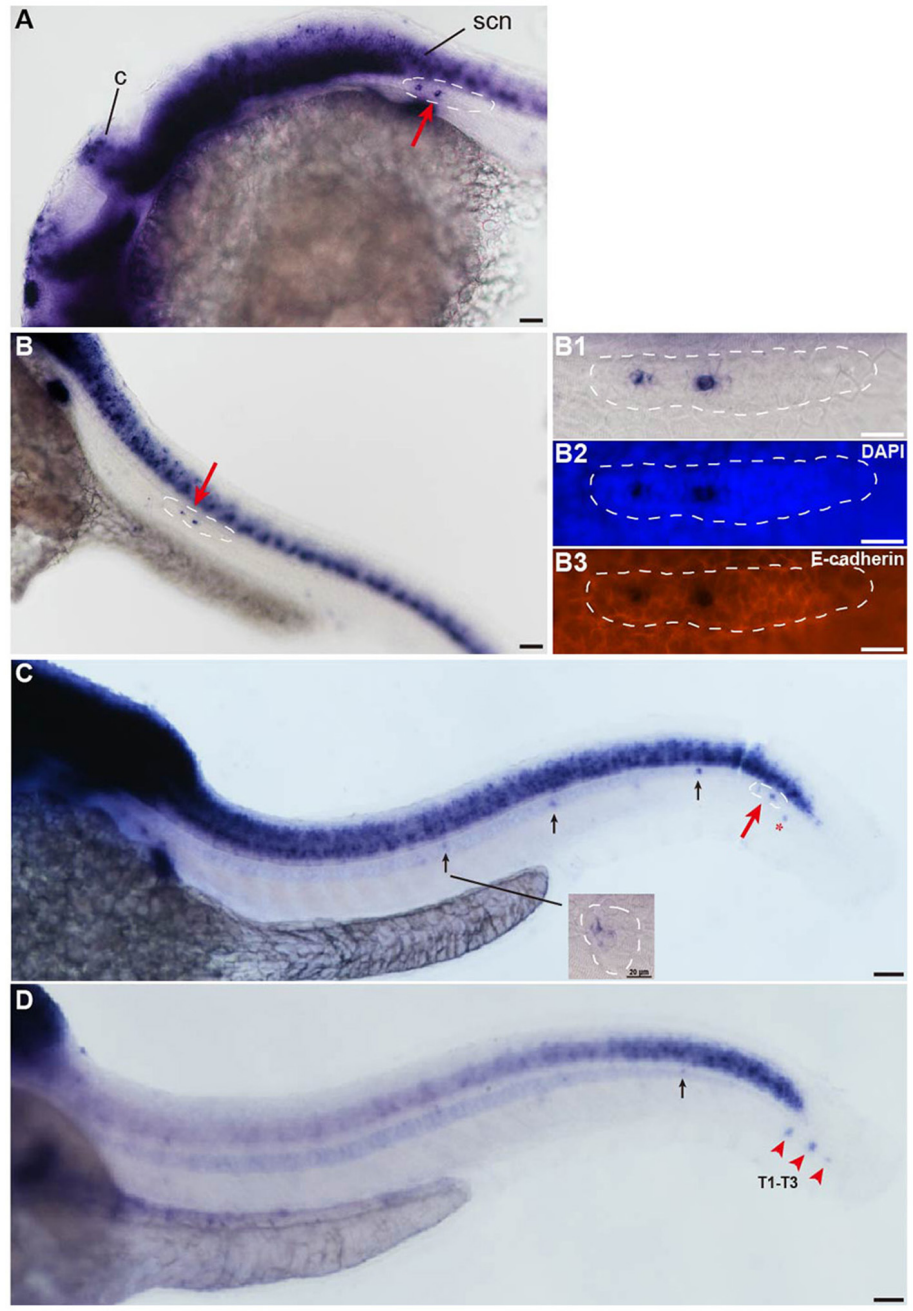
*Insm1a* is expressed in the developing pLL system. **(A–D)** Expression of *insm1a* detected by whole-mount *in situ* hybridization (WISH) in embryos at 24 hpf **(A)**, 30 hpf **(B)**, 48 hpf **(C)**, and 72 hpf **(D)**. *Insm1a* is expressed in the pLL system (Lateral view). **(B1–B3)** The right column of **(B)** shows the pLLp at higher magnification. The primordia are outlined with dotted lines and indicated by red arrows, and the neuromasts are indicated by black arrows. The red arrowheads in **(D)** indicate the terminal neuromasts (T1-T3), and the red asterisk in **(C)** indicates the primordium on the other side of the zebrafish. c, cerebellum; scn, spinal cord neuron. In all figures, scale bars are 50 μm, except the higher magnification image in **(C)**, where the scale bar is 20 μm.

The *insm1a* gene is widely expressed in the developing nervous system during the early stages of embryonic development (Breslin et al., 2003; Forbes-Osborne et al., 2013). WISH experiments in wild-type larvae showed that *insm1a* was strongly expressed in the brain at 24 hpf and could be detected in the pLLp (Figure 1A). From 28 to 48 hpf, zebrafish *insm1a* mRNA was expressed in the pLLp throughout its migration along the body and was expressed in the deposited neuromast cells (Figures 1B,C). Immunohistochemistry for E-cadherin and WISH for *insm1a* confirmed that *insm1a* was specifically expressed in the center of the pLLp of the zebrafish embryos (Figure 1B). At 48 hpf, *insm1a* expression was still present in the pLL system but was beginning to decrease in the neuromasts (Figure 1C). At 72 hpf, *insm1a* mRNA continued to be expressed in the terminal neuromasts with reduced or no expression in the neuromasts along the trunk (Figure 1D). These results suggest that *insm1a* might have an important role in the regulation of embryonic zebrafish pLL development.

### *Insm1a* is required for embryonic pLL morphogenesis

**Figure 2:**
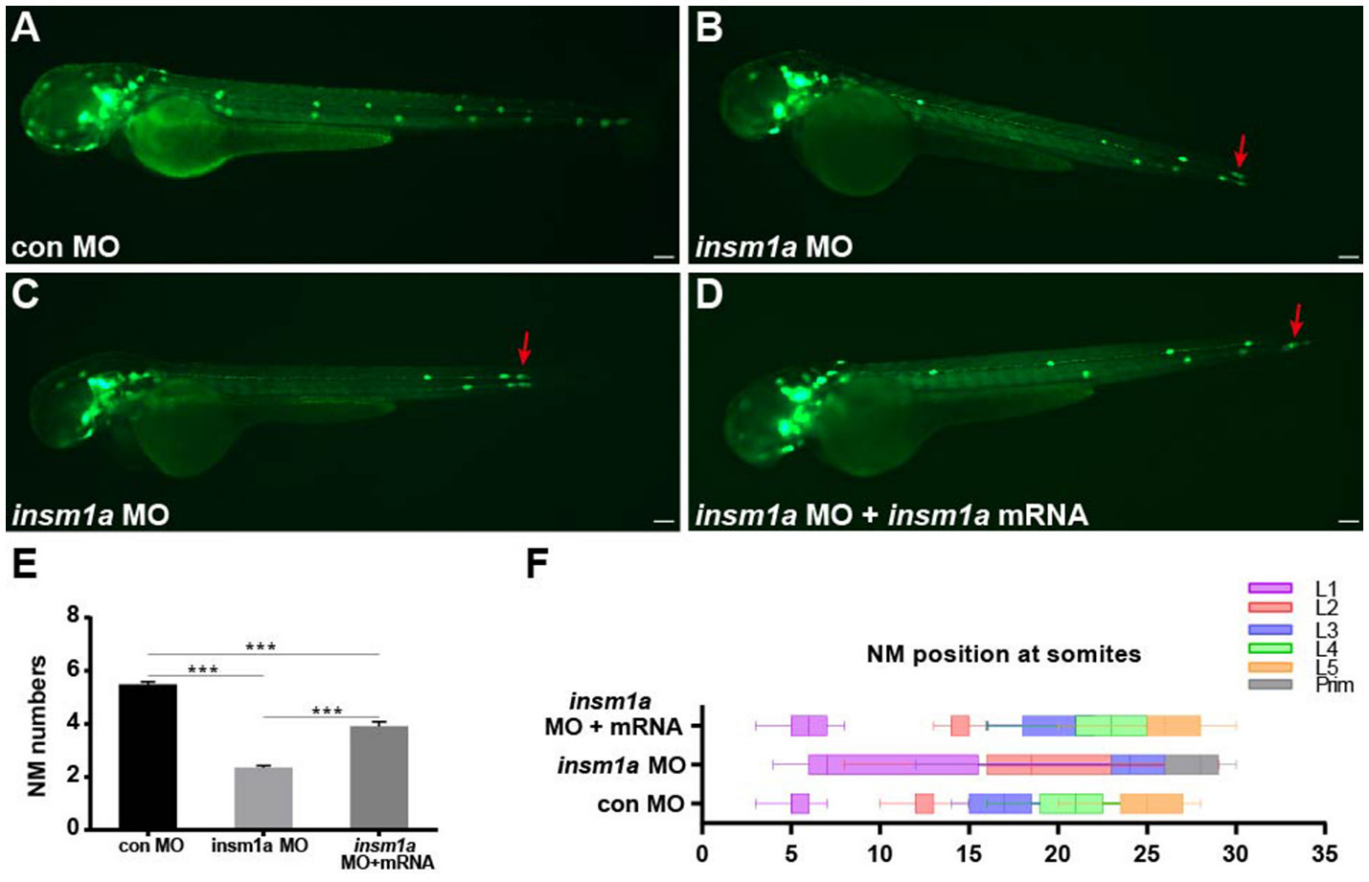
*Insm1a* is required for proper migration of the pLLp and neuromast deposition. **(A–D)** Fluorescent images of *cldnb*:lynGFP embryos at 48 hpf injected with control-MO **(A)**, *insm1a*-MO **(B,C)**, and *insm1a-*MO + *insm1a* mRNA **(D)**. Red arrows indicate the position of the primordium. Scale bars: 100 μm. **(E)** Quantification of the number of neuromasts (NM) along the body at 48 hpf in control-MO (*n* = 25), *insm1a-*MO (*n* = 121), and *insm1a-*MO + *insm1a* mRNA (*n* = 24). ^***^p < 0.001. **(F)** The distribution of deposited neuromasts L1 to L5 in control-MO, *insm1a*-MO, and *insm1a*-MO + *insm1a* mRNA at 48 hpf.

To investigate the function of *insm1a* in pLL morphogenesis,we used a translational antisense morpholino knockdown strategy to inhibit *insm1a*.We first assessed the efficiency of the knockdown by performing western blot on protein extracted from embryos injected with control-MO or *insm1a*-MO. There was a marked reduction of *Insm1a* protein in embryos injected with *insm1a*-MO compared to control-MO (Figure S1A). To exclude the general nonspecific effects of morpholinos linked to *p53* activation, we co-injected embryos with *p53*-MO and *insm1a*-MO in all experiments in this study. Co-injection with control-MO and *p53-*MO resulted in embryos that were indistinguishable from control-MO–injected embryos, suggesting that pLL development was unaffected by loss of p53 (Figures S1B,D), thus control embryos only received control-MO in the following experiments. At 72 hpf, the majority of the *insm1a* morphants showed no obvious differences in terms of overall morphology (Figures S1E,F), although some *insm1a* morphants had slightly smaller heads, slightly smaller eyes, and slight cardiac edema. To evaluate the pLL phenotypes, *tg*(*cldnb*:lynGFP) transgenic embryos that express GFP in the pLLp and the neuromasts were injected with control-MO and *insm1a*-MO. At 48 hpf, the pLL system in control-MO–injected embryos contained an average of 5.48 neuromasts (*n* = 25) in a periodic pattern over the trunk and two or three terminal neuromasts at the tip of the tail (Figures 2A,E). However, the neuromast numbers in the pLL system were significantly reduced in *insm1a-*MO–injected embryos (2.35 ± 0.08 neuromasts; *n* = 121; Figures 2B,C,E). Because pLLp migration was usually slightly delayed at 48 hpf in *insm1a* morphants, we examined the number of neuromasts formed at 72 hpf. Almost all of the primordia had reached the tail-tip where the pLLp normally stops migrating, but neuromast numbers were still drastically reduced (Figure S2).

We next focused on the positioning of neuromasts in the *insm1a* morphants. As illustrated in Figures 2B,C, loss of *insm1a* severely altered the neuromast distribution resulting in an erratic pattern compared with the regularly spaced neuromasts along the trunk of control embryos (Figure 2F) at 48 hpf. In about 55% (*n* = 66/121) of the morphants, the first neuromast (L1) was deposited normally, whereas the following neuromast (L2) was found at a more posterior position with the last two or three neuromasts (L3/L4–L5) often absent (Figures 2B,F). In the remaining morphants (*n* = 55), the L1 position was displaced very posteriorly and the last few neuromasts were often lost (Figures 2C,F). To further validate the phenotype specificity, we co-injected *insm1a* mRNA along with *insm1a*-MO. Co-injection of *insm1a* mRNA partially rescued the pLL defects, with the number of trunk neuromasts recovering to 3.9 (*n* = 24) and the neuromast positioning appearing normal (Figures 2D–F). Taken together, these results suggest that loss of *insm1a* disrupts proper pLL morphogenesis during early zebrafish development.

### *Insm1a* regulates the development of hair cells

**Figure 3:**
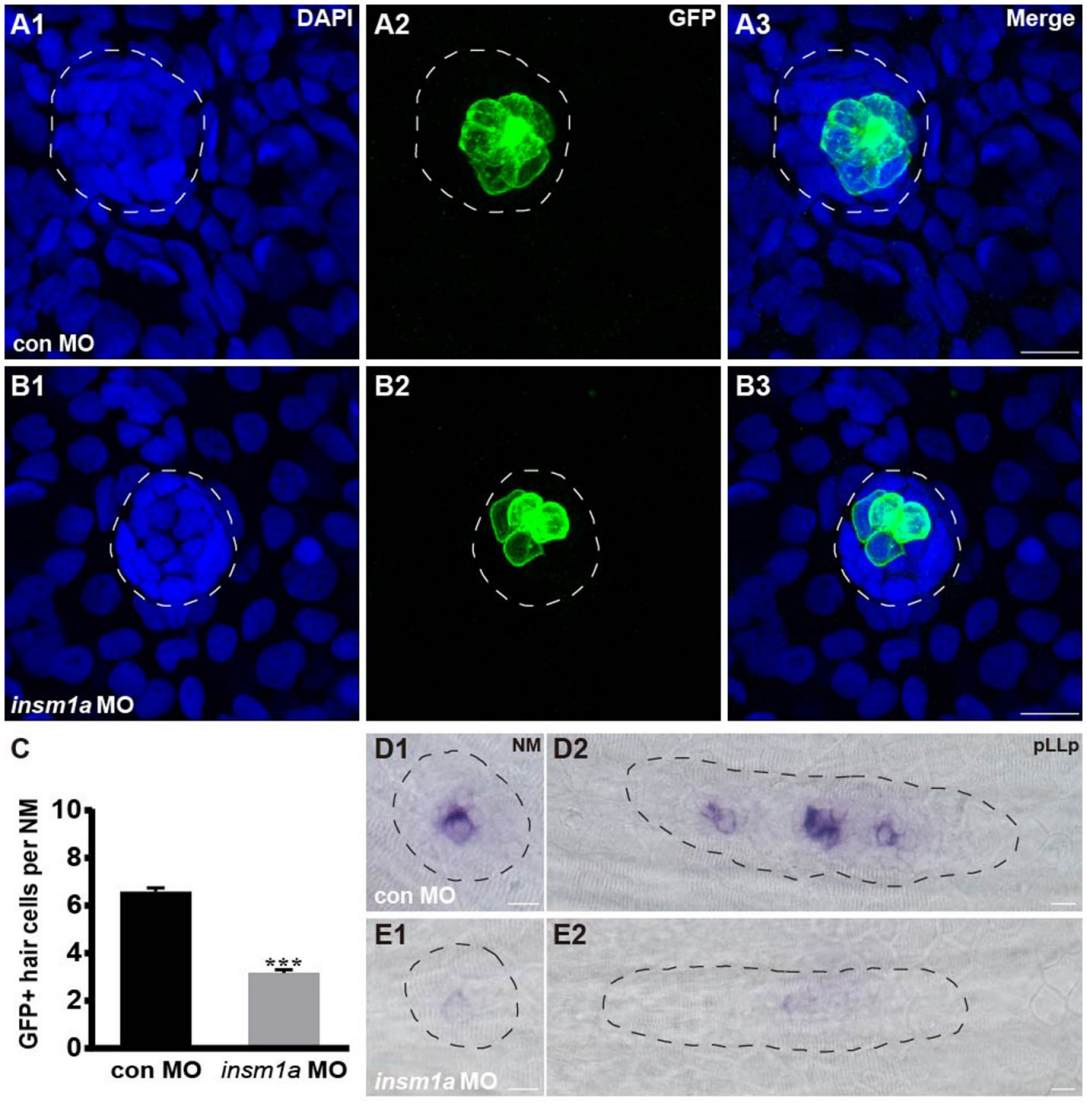
*Insm1a* regulates hair cell formation in the neuromasts. **(A,B)** Representative images of the GFP-expressing hair cells in 72 hpf *Brn3c*:GFP control embryos **(A)** and *insm1a* morphants **(B)**. **(C)** Bar graph of the quantification of the average GFP-positive hair cell numbers per neuromast (NM) for each group. Data are expressed as means ± s.e.m. ^***^p < 0.001. **(D,E)** Lateral views of neuromasts and primordia stained by WISH with an *atoh1a* probe at 30 hpf. The primordium and neuromast are outlined with dotted lines. In all figures, scale bars are 10 μm.

To determine whether the loss of *insm1a* influences hair cell formation, *tg*(*Brn3c*:GFP) transgenic embryos were injected with control-MO or *insm1a*-MO, and anti-GFP antibody was used to label the hair cells in the neuromasts. At 72 hpf, *insm1a* morphants exhibited decreased numbers of GFP-labeled hair cells compared with controls (3.2 ± 0.13 hair cells, *n* = 28 vs. 6.58 ± 0.16 hair cells, *n* = 26, *p* < 0.001; Figures 3A–C). To further investigate the effect of *insm1a* on hair cell precursors in neuromasts, we injected *insm1a*-MO into the wild-type embryos at the one-cell to two-cell stage. At 30 hpf, *insm1a* morphants were collected and subjected to WISH analysis using the probe for the proneural gene *atoh1a*, which is a marker for cells fated to become neuromast hair cells (Itoh and Chitnis, 2001). We observed a reduction in signal for the migrating pLLp and a reduction in the number of deposited neuromasts in *insm1a* morphants (Figures 3E1,E2) compared to controls (Figures 3D1,D2). These data suggest that *insm1a* can regulate *atoh1a* expression and thus promote hair cell formation.

### *Insm1a* regulates cell proliferation in the primordium

**Figure 4:**
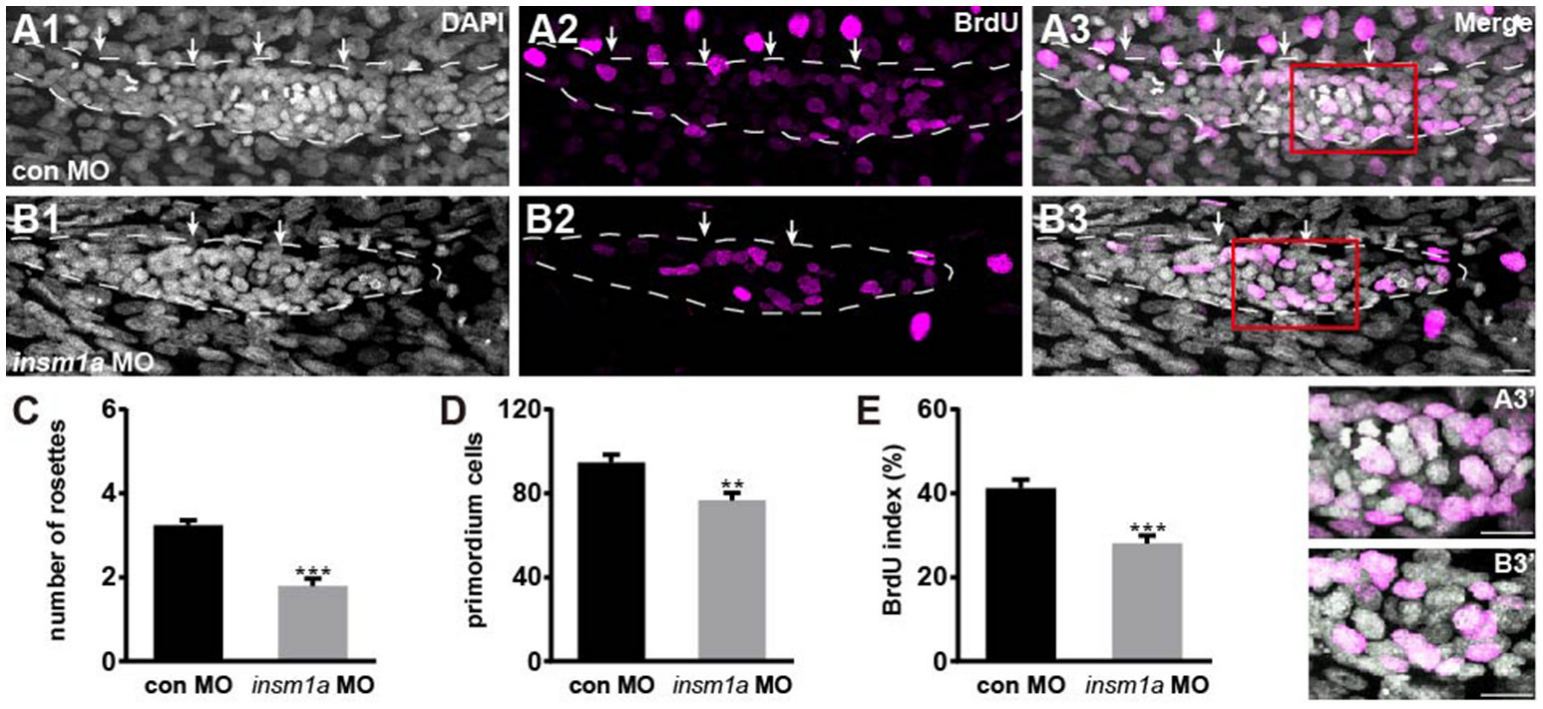
*Insm1a* is required for cell proliferation in the migrating pLLp. **(A,B)** Representative images of the BrdU immunostaining for proliferating cells in the pLLp in controls **(A1–A3)** and *insm1a* morphants **(B1–B3)** at 32 hpf showing that knocking down of *insm1a* impairs rosette assembly and cell proliferation. The primordium is outlined with a dotted line. **(A3′–B3′)** BrdU-positive cells in the rosette at high magnification. White arrows indicate rosettes. Scale bars: 10 μm. **(C–E)** Quantification of rosette numbers **(C)**, cell numbers **(D)**, and BrdU+ primordium cells (BrdU index) **(E)** in controls and *insm1a* morphants. Data are expressed as mean ± s.e.m. ^***^p < 0.001; ^**^p < 0.01.

The number of epithelial rosettes was significantly reduced in *insm1a* morphants at 32 hpf when compared with control primordia, which normally contained two to four rosettes (white arrows in Figures 4A–C). This suggests that *insm1a* is required for rosette assembly in the migrating pLLp. Loss of *insm1a* decreased the size of the pLLp throughout the migration process, and cell counts showed a significant decrease in cell numbers in *insm1a* morphant pLL primordia compared to control pLL primordia (76.5 ± 3.7 cells, *n* = 10 vs. 94.6 ± 3.66 cells, *n* = 14, *p* = 0.0026; Figure 4D). Because previous studies showed that Insm1 is involved in cell proliferation by interacting with cell cycle regulatory proteins (Liu et al., 2006; Zhang et al., 2009), we asked whether reduced cell proliferation is the cause of the reduced cell counts in the primordia of *insm1a* morphants. We performed a bromodeoxyuridine (BrdU) incorporation assay to determine the proliferation status of the pLLp in *insm1a* morphants and control embryos at 32 hpf. There was a significant decrease in the BrdU index in the *insm1a* pLLp compared to controls (28.06 ± 1.92%, *n* = 10 vs. 41.2 ± 2.02%, *n* = 14, *p* = 0.0002; Figures 4A,B,E). To determine if *insm1a* knockdown induces cell death, *insm1a* morphants were allowed to develop until 32 hpf and were analyzed for apoptosis with the TUNEL assay and caspase-3 immunolabeling. No significant apoptosis was detected either in control primordia or in *insm1a* primordia (Figure S3). Taken together, our results indicated that *insm1a* is likely an important regulator of cell proliferation during pLL development.

### *Insm1a* is required for *cxcr12a* and *cxcr4b* expression in the primordium

**Figure 5:**
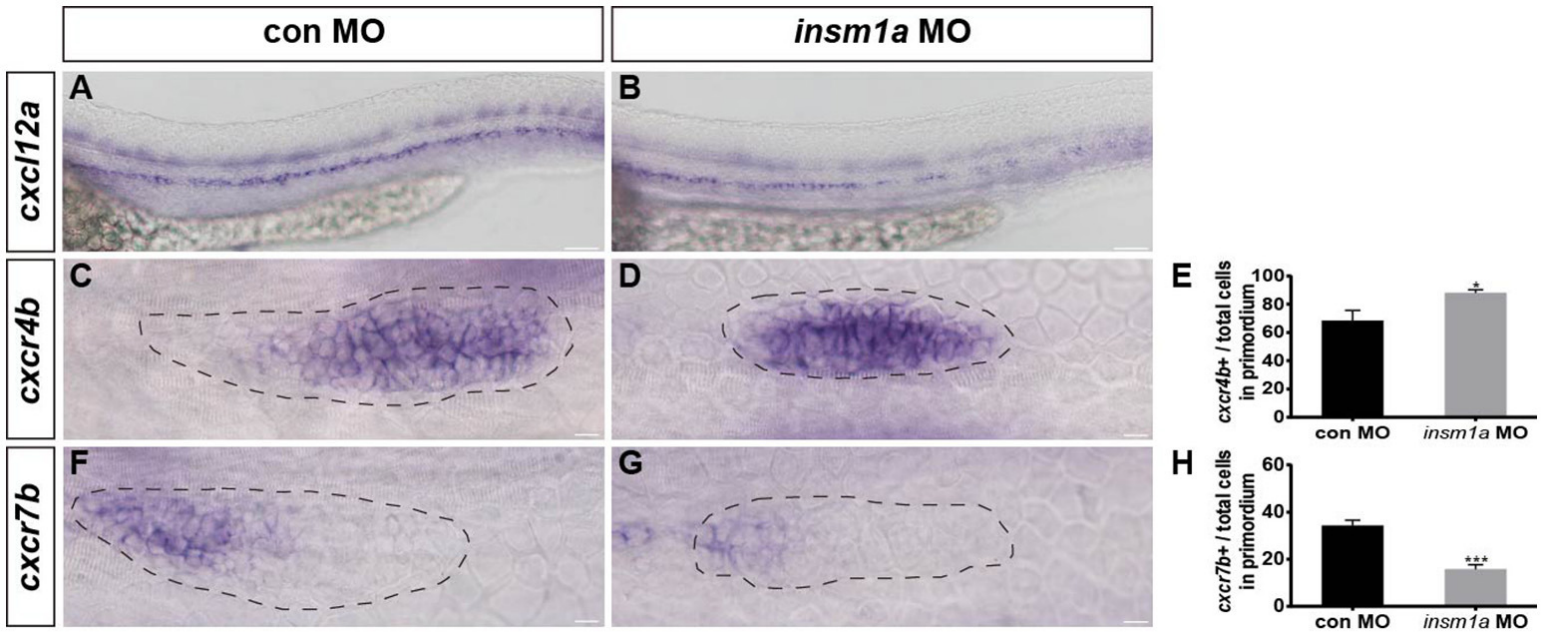
*Insm1a* regulates the expression of chemokine signaling components. WISH was used to detect the expression of *cxcl12a* **(A,B)**, *cxcr4b* **(C,D)**, and *cxcr7b* **(F,G)** in control and *insm1a* morphants at 30–32 hpf. **(E,H)** The ratio of *cxcr4b*-positive cells **(E)** or *cxcr7b*-positive cells **(H)** to total cells in the primordia of controls (black bars) and *insm1a* morphants (gray bars). Data are expressed as mean ± s.e.m. ^*^p < 0.05; ^***^p < 0.001. The *cxcl12a* expression is reduced in *insm1a* morphants, while *cxcr4b* expression is up-regulated and *cxcr7b* expression is down-regulated in *insm1a* morphants compared to controls. The primordium is outlined with a dotted line, and the anterior is to the left. Scale bar: 50 μm **(A,B)** and 10 μm **(C–F)**.

In zebrafish, chemokine signaling pathways play central roles in the directional migration of the pLLp (Haas and Gilmour, 2006). The chemokine ligand *cxcl12a* is expressed along the horizontal myoseptum of zebrafish, and the receptors *cxcr4b* and *cxcr7b* are normally polarized to the leading and trailing zone of the migrating primordium, respectively (Valentin et al., 2007). Given the severe primordium migration defects in *insm1a* morphants, we asked whether *Insm1a* functions in the control of pLL morphogenesis by regulating chemokine signaling. We examined the expression of a suite of region-specific chemokine markers during the early developmental stage, and WISH analysis showed that the expression of the myoseptum-expressed *cxcl12a* was altered in *insm1a* morphants compared to controls (Figures 5A,B). The *insm1a*-MO embryos showed a broad expansion of *cxcr4b* expression (Figures 5C–E), whereas the expression domains of *cxcr7b* were reduced in the primordia of these embryos at 30–32 hpf (Figures 5F–H). Thus, we speculated that *Insm1a* might regulate pLL morphogenesis in part by modulating chemokine gene expression and thus controlling primordium migration.

### Loss of *insm1a* affects Wnt and Fgf signaling in the primordium

**Figure 6:**
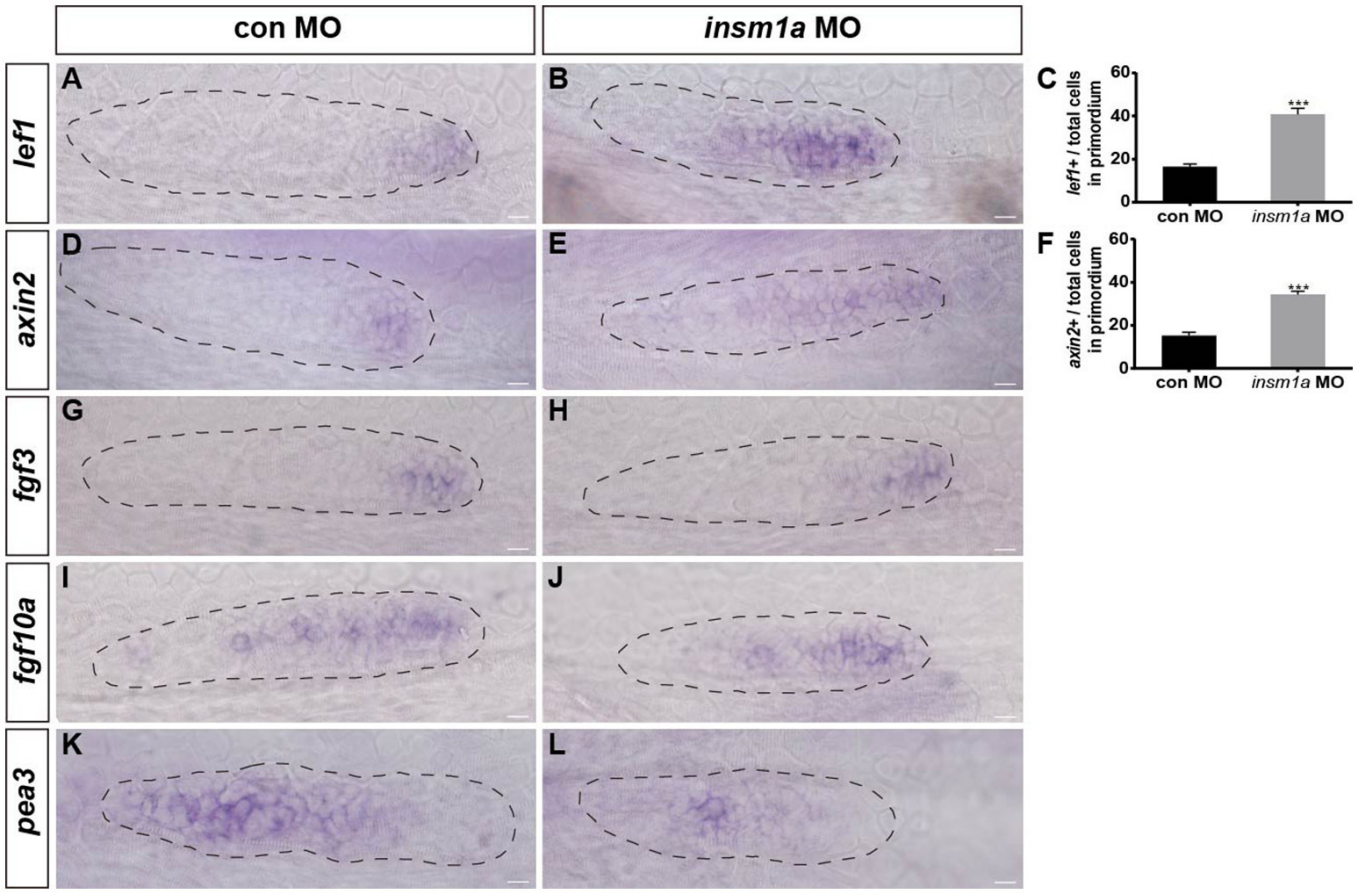
*Insm1a* regulates the expression of Wnt and Fgf signaling components. Expression of the Wnt and Fgf target genes *lef1* **(A,B)**, *axin2* **(D,E)**, *fgf3* **(G,H)**, *fgf10a* **(I,J)**, and *pea3* **(K,L)** in control embryos and in *insm1a* morphants at 30–32 hpf. **(C,F)** The ratio of *lef1*-positive cells **(C)** or *axin2*-positive cells **(F)** to total cells in the primordia of controls (black bars) and *insm1a* morphants (gray bars). Data are expressed as mean ± s.e.m. ^***^p < 0.001. In *insm1a* primordia, the expression domains of *lef1* and *axin2* expand, whereas *fgf3* and *fgf10a* expression is normal but *pea3* is reduced. The primordium is outlined with a dotted line, and the anterior is to the left. Scale bar: 10 μm.

Wnt/β-catenin plays central roles in regulating the migration and proliferation of the primordium during early pLL development (Aman and Piotrowski, 2008; Aman et al., 2011), and Wnt/β-catenin signaling is thought to restrict the localized expression pattern of the chemokine receptors *cxcr4b* and *cxcr7b* to the leading and trailing edges of the primordium, respectively (Aman and Piotrowski, 2008). To determine whether *Insm1a* functions in the control of pLL morphogenesis through regulation of the Wnt/β-catenin signaling pathway, we first examined the expression of *lef1*, a direct Wnt target gene, whose expression is normally restricted to the leading-edge cells in the migrating primordium, as shown in control embryos (Figure 6A). In contrast, in *insm1a* morphants, the expression of *lef1* significantly expanded, although it was still restricted to the leading-edge cells of the primordium compared to controls (Figures 6B,C). Likewise, the expression area of *axin2* was also expanded in the primordium of *insm1a* morphants as compared to controls (Figures 6D–F). These findings indicate that the *insm1a* gene is a negative regulator of Wnt/β-catenin signaling in the control of pLL development in zebrafish. Because the correct patterning and migration of the pLLp depends on the interaction between Wnt/β-catenin and Fgf signaling, we next asked whether the pLL phenotypes observed in *insm1a* morphants were also associated with the Fgf signaling pathway. WISH showed that loss of *insm1a* had no significant effect on the expression levels of *fgf3* (Figure 6H) or *fgf10a* (Figure 6J) compared to controls (Figures 6G,I). In contrast, embryos injected with *insm1a*-MO showed markedly reduced expression of *pea3*, a Fgf target gene, in the primordium (Figures 6K,L).

## Discussion

In the developing mouse ear, *insm1* is expressed in the delaminating and delaminated neuronal progenitors and promotes their proliferation along with SVG neurogenesis beginning at embryonic day (E)10.5 (Lorenzen et al., 2015). *Insm1* mutant mice have fewer cell divisions and thus produce fewer neurons within the otic ganglia at developmental stages after E10.5. However, whether *insm1* is expressed in the auditory organs of other model organisms, and whether it plays a role in driving the specific differentiation of these sensory organs, are not known. In this study, we identify the importance of *Insm1a* function in normal pLL development in zebrafish. We showed that *insm1a* was expressed in the embryonic pLLp and deposited neuromasts, implying that *insm1a* might play important roles in many aspects of pLL development. Indeed, using morpholino antisense technology, our findings indicated that *insm1a* not only regulated pLL formation but also contributed to the differentiation of hair cells. Furthermore, we showed that *insm1a* is required for the normal expression patterning of chemokine, Wnt/β-catenin, and Fgf signaling in the primordium. Thus, our findings identify transcription factor *Insm1a* as a novel regulator in pLL development.

In zebrafish, *insm1a* is expressed throughout the developing nervous system, including the forebrain, midbrain, hindbrain, olfactory placode, cerebellum, and retina, and it is an important player in early embryonic neurogenesis (Lukowski et al., 2006). Our WISH analysis demonstrated that zebrafish *insm1a* mRNA was transiently expressed in the embryonic pLL system, including the migrating primordium and the newly deposited neuromasts, providing new lines of evidence for a role for *insm1a* in normal sensory organ development. The timing of peak *insm1a* expression in the zebrafish pLL system corresponds to the period of primary pLL formation. The signal for *insm1a* was significantly reduced in the neuromasts spaced along the trunk later in development when these neuromasts mature into mechanosensory neuromasts. Our findings are consistent with reports that the *insm1* transcript is transiently expressed in the neuronal progenitors and nascent neurons throughout the developing nervous system and is dramatically decreased after birth (Duggan et al., 2008; Farkas et al., 2008).

In order to investigate the role of *insm1a* in pLL development, we used a translation-blocking antisense morpholino to knockdown *insm1a* expression in zebrafish. Our results demonstrated that *insm1a* gene knockdown resulted in severe pLL developmental defects. Because *insm1a* morphants did not exhibit significant morphological abnormalities during early development, the pLL defects in the morphants are probably due to deficiency of *insm1a*. In *insm1a*-deficient embryos, we observed fewer cells in the migrating primordium when compared with controls. We performed a TUNEL assay and caspase-3 immunohistochemistry to assess the level of cell death in control embryos and *insm1a* morphants. No significant changes were observed in the *insm1a* morphants, demonstrating that the decrease in cell numbers is unlikely to be the result of increased apoptosis. Another potential explanation for the reduced cell numbers in *insm1a* morphants is that loss of *insm1a* might alter cell cycle progression. We next examined the proliferation level in the pLLp by using the BrdU incorporation assay for labeling S-phase cells. We found fewer proliferating cells in the *insm1a* primordia compared to controls. Our results suggest that *Insm1a* is involved in cell cycle progression. As a transcription factor, *Insm1a* has been shown to be a critical regulator in the transcriptional network that mediates various cellular processes such as cell proliferation, cell specification, and cell fates (Liu et al., 2006; Farkas et al., 2008; Wildner et al., 2008; Zhang et al., 2009). For example, *Insm1a* has been implicated in regulating the transcription of cell cycle genes in the mouse neocortex (Farkas et al., 2008), the secondary sympathetic ganglia (Wildner et al., 2008), and the developing and regenerating zebrafish retina (Ramachandran et al., 2012; Forbes-Osborne et al., 2013). Previous reports have shown that Insm1 is involved in modulating transcriptional activity through recruitment of cyclin D1 and histone deacetylases (Liu et al., 2006). Cyclin D1 plays an important role in transcriptional regulation through its association with CDK4 (Sherr, 1996), and cyclin D1-deficient mice have reduced body size and suffer from neurological impairment (Sicinski et al., 1995). Whether the molecular mechanism of *insm1* transcriptional activity in cell cycle functions is due to the recruitment of cyclin D1 or other unknown major components remains unclear and will be investigated in future studies.

The process of pLLp migration is mediated by a network of signaling pathways, including chemokine, canonical Wnt/β-catenin, and Fgf signaling. Among these, chemokine signaling is important in guiding the directed collective migration of the pLLp (David et al., 2002; Li et al., 2004; Dambly-Chaudiere et al., 2007). The chemokine receptors *cxcr4b* and *cxcr7b* are expressed in the leading and trailing zones of the primordium, respectively, and their ligand *cxcl12a* is expressed along the horizontal myoseptum of the zebrafish (David et al., 2002; Haas and Gilmour, 2006; Valentin et al., 2007). Knockdown of *cxcl12a* or its receptor *cxcr4b* or *cxcr7b* leads to strong defects in primordium migration (David et al., 2002; Li et al., 2004; Haas and Gilmour, 2006; Dambly-Chaudiere et al., 2007; Valentin et al., 2007). To understand the effects of the loss of *insm1a* on the chemokine signaling pathway, we examined the expression of *cxcl12a, cxcr4b*, and *cxcr7b*. We found that *insm1a* knockdown led to reduced *cxcl12a* expression along the myoseptum and led to an expansion of *cxcr4b* expression into the trailing part of the pLLp. It also led to a loss of *cxcr7b* expression, although this was not completely abolished compared with control embryos. Our findings show that *insm1a* is involved in the regulation of chemokine signaling in the primordium during development. It has been shown previously that inactivation of Cxcl12a signaling by *cxcl12a*-MO results in a significant expansion of *cxcr7b* expression (Gamba et al., 2010a). However, the expanded expression of *cxcr7b* never extended to the anteriormost cells of the primordium, suggesting that other factors can regulate *cxcr7b* independently of Cxcl12a/cxcr4 signaling (Gamba et al., 2010a). A marked decrease in the expression of *cxcr4b* in the absence of *cxcl12* was also observed (Gamba et al., 2010a). Interestingly, *cxcr4b* still exists in the leading domain, even if the pLLp migrates away from its normal migratory path in *cxcl12a* morphants, suggesting that expression of *cxcr4b* might be under the direct control of other important signaling pathways, perhaps Wnt/β-catenin signaling. The disruption of *cxcr4b* and *cxcr7b* expression in *insm1a*-MO embryos might reflect a direct requirement for *insm1a in* Cxcl12a/cxcr4 signaling or might be a consequence of some other ectopic signaling expression in the primordium. It would be important to verify these proposals in the future by genetic approaches (e.g., inactivation of *cxcl12a, cxcr4b*, or *cxcr7b*) in combination with other approaches such as next-generation sequencing and bioinformatics analysis.

Proper pLLp migration also depends on the Wnt/β-catenin and Fgf signaling feedback system (Aman and Piotrowski, 2008, 2009, 2011). Wnt/β-catenin signaling is active in the leading region of the primordium, where it controls the expression of the Wnt targets *lef1* and *axin2* and regulates multiple aspects of pLL development, including primordium migration, proneuromast formation, and cell proliferation in the primordium (Aman and Piotrowski, 2008; Gamba et al., 2010b; Aman et al., 2011; McGraw et al., 2011; Valdivia et al., 2011). Wnt/β-catenin signaling controls primordium migration by coordinating the localized expression of chemokine receptors *cxcr4b* and *cxcr7*. Wnt activity confines *cxcr7b* expression to the trailing part of the pLLp and is required for maintenance of normal *cxcr4b* expression in the leading part of the pLLp (Aman and Piotrowski, 2008; McGraw et al., 2011; Breau et al., 2013). Activation of the Fgf signaling pathway in the trailing zone of the primordium triggers morphogenesis of the apically constricted rosettes within the primordium and restricts the activation of Wnt activity to the leading zone. Wnt activation, in turn, is necessary for the expression of the Fgf ligands *fgf3* and *fgf10a* in the leading zone of the primordium (Lecaudey et al., 2008; Nechiporuk and Raible, 2008). Activation of the Wnt pathway and/or loss of Fgf signaling results in *cxcr7b* downregulation and *cxcr4b* expansion, which contribute to aberrant pLL development, including disruptions in rosette organization and proneuromast deposition (Aman and Piotrowski, 2008; Nechiporuk and Raible, 2008). It is informative to compare these results with the expression and function of Insm1a. We investigated the effects of loss of *insm1a* expression on Wnt/Fgf signaling and found that the expression of *lef1* and *axin2* were markedly increased in the *insm1a* morphant primordia. To investigate the effects of *insm1a* expression on Fgf signaling, we analyzed the expression of *fgf3, fgf10a*, and *pea3*, which are necessary for proper patterning and migration of the primordium and for neuromast formation and deposition (Aman and Piotrowski, 2008; Lecaudey et al., 2008; Nechiporuk and Raible, 2008). We found that the expression of both the *fgf3* and *fgf10a* genes appeared unchanged in *insm1a* morphants, but there was a marked reduction in *pea3* expression. However, it is still unclear whether *insm1a* is directly or indirectly involved in regulating Wnt/Fgf signaling in the pLLp, and the detailed mechanisms await further investigation.

We have shown here that zebrafish *insm1a* mRNA was transiently expressed during the entire pLLp migration process. Because E-cadherin immunohistochemistry and WISH with an *insm1a* antisense probe showed that *insm1a* was not localized to the leading or trailing cells but was specifically present in the central part of the primordium of the zebrafish embryos, we hypothesized that *Insm1a* is required for rosette assembly and regulates hair cell formation during pLL development. To test this hypothesis, we knocked down *insm1a* expression and showed that loss of *insm1a* significantly reduced the numbers of rosettes in the primordium of *insm1a* morphants compared to controls. As a consequence, the final pattern of pLL was aberrant with very few neuromasts being deposited from the primordia and those that were deposited lacking organized rosettes. Neuromast maturation is another important aspect of the development of the pLL system, and in control embryos the numbers of hair cells in the pLL neuromast increased over time. When we injected *insm1a*-MO into the *Brn3c*:GFP transgenic line, which express GFP in the neuromast hair cells, we observed a significant decrease in the number of hair cells. Our findings also support a recent study documenting the function of Insm1 in delaminating progenitors, nascent neurons, and differentiating outer hair cells of the embryonic mouse inner ear (Lorenzen et al., 2015). Deletion of *insm1* resulted in fewer SVG neurons during otic neurogenesis, suggesting that Insm1 plays a functional role in the differentiation of the nascent neurons. However, that study did not report any obvious abnormalities in the differentiation of hair cells upon the loss of *insm1*. This might be partly due to the embryonic lethality of the *insm1*-null mouse. In our study, because the *insm1a* transcript was expressed transiently in neuromasts during the early developmental stages in zebrafish, we hypothesized that the effect of *insm1a* knockdown on hair cell formation is probably a secondary consequence of the absence of organized rosettes in the primordium. Previous studies have shown that *atoh1a* is typically restricted to central cells in the pLLp, which gives central cell clusters the ability to develop into sensory hair cells (Lecaudey et al., 2008; Nechiporuk and Raible, 2008). In our study, we observed that *atoh1a* expression was lost in some hair cell clusters of both the migrating pLLp and neuromasts in *insm1a* morphants, suggesting that *insm1a* might regulate hair cell formation by regulating *atoh1a* expression.

In summary, our study suggests an important role for the transcription factor *Insm1a* in zebrafish pLL development. We show that *Insm1a* is required for the proliferation of pLLp cells and the development of hair cells. We further reveal the association between *Insm1a* and Wnt signaling in regulating pLL formation. Further analysis is needed to determine whether *Insm1a* is also involved in other signaling pathways and whether it regulates the expression of other transcription factors involved in primordium migration and neuromast differentiation. Identifying the direct targets of *Insm1a* during pLL development will provide helpful insights into the underlying mechanisms of hearing development and hearing-related diseases.

## Author contributions

YH, RC, HL, and DL conceived and designed the work. YH and RC wrote the manuscript. YH, XL, and FQ performed the zebrafish experiments. HL, XL, FQ, and DL performed data analyses. All authors discussed the data, and all authors reviewed the manuscript.

### Conflict of interest statement

The authors declare that the research was conducted in the absence of any commercial or financial relationships that could be construed as a potential conflict of interest.

## Supplementary material

Figure S1 **(A)** Western blot detection of *Insm1a* protein in embryos injected with 6 ng *insm1a* antisense morpholino or 6 ng control morpholino showing a marked reduction of *Insm1a* protein in the *insm1a* morphants. The experiment was repeated two times. **(B,C)** Fluorescent images of *cldnb*:lynGFP embryos at 48 hpf injected with control-MO (con MO) **(B)** and con MO + *p53* MO **(C)**. Scale bars: 100 μm. **(D)** Quantification of the number of neuromasts along the body at 48 hpf in con MO (*n* = 20) and con MO + *p53* MO (*n* = 20). There was no significant difference (*p* > 0.05). **(E,F)** Transmitted light images of 72 hpf control morpholino-injected embryo (con MO) **(E)** or *insm1a* morphants (*insm1a* MO) **(F)**. The overall morphology of the *insm1a* morphants was indistinguishable from controls. Scale bars: 100 μm.

Figure S2 *Insm1a* knockdown shows a reduction in the number of pLL neuromasts. **(A,B)** Fluorescent images of *cldnb*:lynGFP embryos at 72 hpf injected with control-MO and *insm1a*-MO. Scale bars: 100 μm. **(C)** Quantification of the number of neuromasts along the body at 72 hpf in control-MO (*n* = 20) and *insm1a*-MO (*n* = 38). ^***^*p* < 0.001.

Figure S3 Loss of *insm1a* has no significant effect on cell death. **(A,B)** TUNEL assay on sections from a control **(A)** and *insm1a* morphant **(B)** at 32 hpf. **(C,D)** Representative images of cleaved caspase-3 immunohistochemistry in primordia from a control **(C)** and *insm1a* morphant **(D)**. No significant difference in the number of apoptotic cells in the *insm1a* morphant was detected compared to controls. Scale bar: 100 μm **(A,B)** and 10 μm **(C,D)**.
