# Supplementary figures and images for: Insm1a Is Required for Zebrafish Posterior Lateral Line Development
Source: Front Mol Neurosci. 2017 Aug 2;10:241. doi: 10.3389/fnmol.2017.00241 (PMC5539400; doi:10.3389/fnmol.2017.00241)

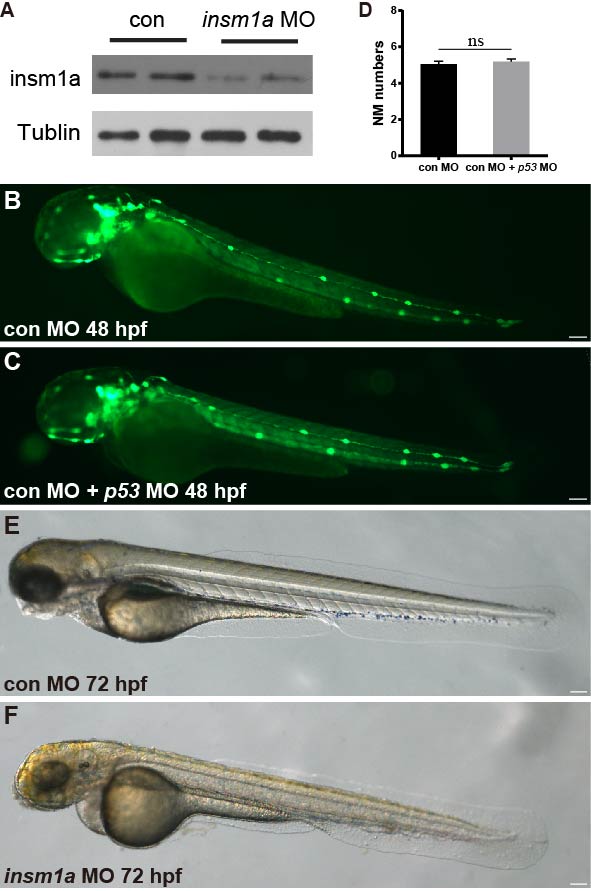

Supplement: Figure S1 — (A) Western blot detection of Insm1a protein in embryos injected with 6 ng insm1a antisense morpholino or 6 ng control morpholino showing a marked reduction of Insm1a protein in the insm1a morphants. The experiment was repeated two times. (B,C) Fluorescent images of cldnb:lynGFP embryos at 48 hpf injected with control-MO (con MO) (B) and con MO + p53 MO (C). Scale bars: 100 μm. (D) Quantification of the number of neuromasts along the body at 48 hpf in con MO (n = 20) and con MO + p53 MO (n = 20). There was no significant difference (p > 0.05). (E,F) Transmitted light images of 72 hpf control morpholino-injected embryo (con MO) (E) or insm1a morphants (insm1a MO) (F). The overall morphology of the insm1a morphants was indistinguishable from controls. Scale bars: 100 μm. [file Image1.JPEG]

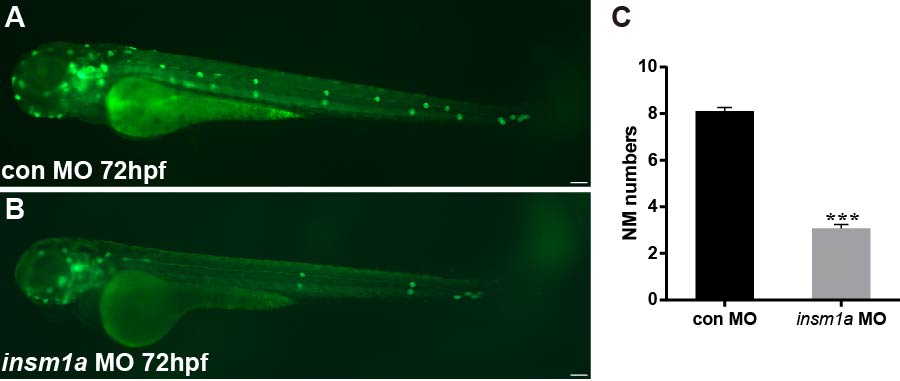

Supplement: Figure S2 — Insm1a knockdown shows a reduction in the number of pLL neuromasts. (A,B) Fluorescent images of cldnb:lynGFP embryos at 72 hpf injected with control-MO and insm1a-MO. Scale bars: 100 μm. (C) Quantification of the number of neuromasts along the body at 72 hpf in control-MO (n = 20) and insm1a-MO (n = 38). ***p < 0.001. [file Image2.JPEG]

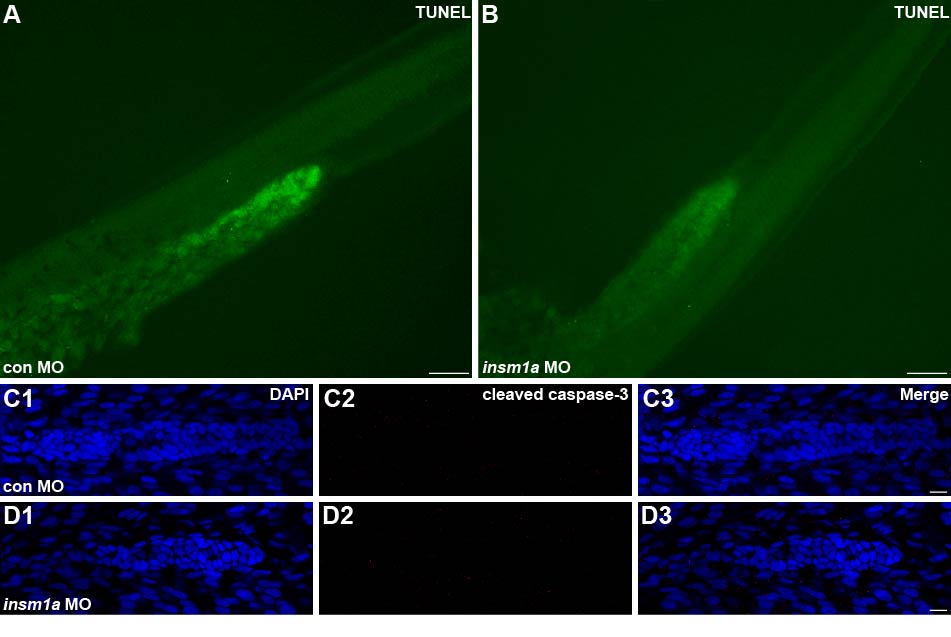

Supplement: Figure S3 — Loss of insm1a has no significant effect on cell death. (A,B) TUNEL assay on sections from a control (A) and insm1a morphant (B) at 32 hpf. (C,D) Representative images of cleaved caspase-3 immunohistochemistry in primordia from a control (C) and insm1a morphant (D). No significant difference in the number of apoptotic cells in the insm1a morphant was detected compared to controls. Scale bar: 100 μm (A,B) and 10 μm (C,D). [file Image3.JPEG]
